# A New High-Resolution Spectral Approach to Noninvasively Evaluate Wall Deformations in Arteries

**DOI:** 10.1155/2014/606202

**Published:** 2014-02-13

**Authors:** Ivonne Bazan, Carlos Negreira, Antonio Ramos, Javier Brum, Alfredo Ramirez

**Affiliations:** ^1^ESIME Zacatenco, Instituto Politécnico Nacional (IPN), Avenue IPN, s/n, 07738 Mexico, DF, Mexico; ^2^Departamento de Materiales, Facultad de Ciencias, Universidad de la Republica, 14200 Montevideo, Uruguay; ^3^Institute of Physical & Information Technologies, CSIC, Serrano 144, 28006 Madrid, Spain

## Abstract

By locally measuring changes on arterial wall thickness as a function of pressure, the related Young modulus can be evaluated. This physical magnitude has shown to be an important predictive factor for cardiovascular diseases. For evaluating those changes, imaging segmentation or time correlations of ultrasonic echoes, coming from wall interfaces, are usually employed. In this paper, an alternative low-cost technique is proposed to locally evaluate variations on arterial walls, which are dynamically measured with an improved high-resolution calculation of power spectral densities in echo-traces of the wall interfaces, by using a parametric autoregressive processing. Certain wall deformations are finely detected by evaluating the echoes overtones peaks with power spectral estimations that implement Burg and Yule Walker algorithms. Results of this spectral approach are compared with a classical cross-correlation operator, in a tube phantom and “in vitro” carotid tissue. A circulating loop, mimicking heart periods and blood pressure changes, is employed to dynamically inspect each sample with a broadband ultrasonic probe, acquiring multiple A-Scans which are windowed to isolate echo-traces packets coming from distinct walls. Then the new technique and cross-correlation operator are applied to evaluate changing parietal deformations from the detection of displacements registered on the wall faces under periodic regime.

## 1. Introduction and Background

The advent of vascular diseases, such as hypertension and atherosclerosis, is associated with significant alterations in the physical properties of arterial vessels [1, 2]. They originate coronary and cerebrum-vascular accidents are currently the more common causes of human morbidity and mortality. The evaluation of the arterial biomechanical behavior is related to the assessment of three representative indices: arterial compliance, arterial distensibility, and arterial stiffness. Elasticity is the most important mechanical property of the arterial walls, whose nature is strictly nonlinear. In this analytical context, the responses of elastin and collagen fibers (passive constituent elements of the arterial wall) are related to the stress level applied to the wall. Concerning this, appropriate ultrasonic tools are required to analyze the temporal dynamics of the echo-signals involved, in order to characterize the whole phenomenon.

The determination of alterations in the arterial structure and in elastic properties that occur in the early stages of the arterial diseases could allow implementing actions to avoid the disease progression and development. Unfortunately, the diagnostic techniques nowadays available only allow detecting the structural and biomechanical alterations already in advanced stages of the disease, when it is widely disseminated (regional alterations). Consequently, to generate diagnostic tools to detect early alterations in local zones of the arterial walls is a compulsory necessity in the field of preventive medicine.

In this diagnosis problem, the early detection of silent atherosclerosis is a possibility for detecting asymptomatic patients with high potential for cardiovascular complications [3]. In this regard, it is known that atherosclerosis progresses over many years in asymptomatic and silent way and often in its first clinical manifestation is expressed as a cardiovascular complication, and it could even produce the patient's death. Recent advances in noninvasive techniques applied to arteries in humans have shown the potential capability to detect various abnormalities in the function or in the structure of the arterial walls representing early atherosclerosis.

### 1.1. Brief Review of Research Methods to Detect Arterial Alterations

Currently, it is well known that the major markers that indicate the existence of atherosclerotic disease include thickening of the arterial wall (which is related, actually, to the intimae-media thickness), arterial calcification, arterial stiffness and endothelial dysfunction [3]. The determination of mechanical alterations (in thickening and stiffness, of the walls) usually includes, as a first step, an estimation of geometrical deformations in the wall thickness along the cardiac cycle, from the analysis of ultrasonic information acquired by echo-interrogation. For obtaining a good estimation, this type of thickening measurements (improving the data provided by typical medical imaging units) must be made by applying a variety of signal processing methods (generally, of a more general purpose). They operate mainly in time and phase domains and have been described in the literature, specially along the two last decades [4–13]. These methods use a number of techniques with their successive variants and improvements: various autocorrelation types, time echo-tracking methods, quadrature demodulations, RF and complex cross-correlations, phase tracking, and so forth. Some of them are extensions coming from velocity Doppler estimation procedures adapted to this finality. A good and complete review of comparative performances in several of these options can be found in [9], where particular limitations in some cases (of statistical origin), as certain systematic bias, are analyzed in detail, for example, those derived of integrating velocity estimates, dependences on changes of the center RF, or well, parabolic, or gaussian interpolations.

Finally, by analyzing the relative deformations of the wall thickness, under pressure perturbations into the blood vessel [6, 8], an estimation of the elastic modulus EM can be calculated in arterial points:

(1) $$\begin{array}{c} \text{EM}=\frac{\left(P_{\max}-P_{\min}\right)}{\left(\text{Df}/{\text{Th}}_{0}\right)}, \end{array}$$

where *P* are extreme blood pressures; Th_0_ is arterial wall thickness at the end of diastole period; and Df the maximum wall variation or total deformation in thickness.

The processing of B-mode ultrasonic imaging is used to estimate the intima-media thickness, but does not allow a very good local determination, because initial data are related to axial resolutions in typical medical imaging. In parallel and complementary way, the arterial stiffness (a marker of the sclerotic component of atherosclerosis) can be assessed by using measurements of arterial elasticity, well by means of the classical pulse wave velocity method, or well with a more recent estimate of arterial stiffness by ultrasound systems tracking movements of arterial walls during the cardiac cycle [14]. These last options are in experimental stages, and there is a current need for highly sensitive tools detecting localized changes in wall biomechanical properties, particularly for evaluating “local” changes in small wall zones suffering early alteration stages.

There is an extensive research [15–20] conducted to the developing of phase tracking methods, for measuring the changing wall thickness during the heartbeat, by estimating velocities in arterial walls. In this context, additional efforts improving ultrasonic techniques in order to detect small deformations localized at particular arterial points are being made in time and phase domains [21–25], to assess walls elasticity, by speckle interferometry; here, arterial behavior in radial direction is studied from displacements of four wall interfaces, under variations in pressure applied from internal liquid.

In this paper, a new ultrasonic technique, intended to characterize local alterations in arterial walls, is proposed. It works directly in the frequency domain, in particular in the range of high-order echo-overtones. Its technological implementation, of rather low-cost nature, integrates uniquely: a broadband piezoelectric transducer in MHz range, a pulsed electronic transceiver, and a spectral processing stage to manage echo-signal overtones. Involved signal processing is based on improving high-resolution calculation algorithms for the location of the peak frequency in the harmonic band selected into the power spectral density (PSD) curve of acquired wall echo-traces. For making this frequency location, two parametric autoregressive (AR) methods are employed [26–28].

Some results, obtained with this technique, are here compared in well-controlled multipulse echo patterns, with other results obtained using an approach based on one of the previous alternative techniques conventionally used for artery analysis, the basic time cross-correlations (TCC) of ultrasonic echoes coming from wall interfaces in distinct cycle points. There are a number of works that successfully use the cross-correlation operator to detect moving reflectors or internal properties into biological tissues [4, 5, 7, 9, 26, 29]. And other works related directly to this arterial problem use this same TCC time operator in an elastographic context [12, 23–25].

The operator TCC was chosen as a reference in the analysis of our approach for its simple implementation to have results independent of the actual center frequency value [9] and besides because it was proved by us that this option provides a reasonable spatial resolution just in this type of biomedical applications [26, 30].

## 2. Spectral Parametric Technique Improving Resolution to Estimate Geometrical Deformations in Wall Thickness

The aim of this paper is to show advances and laboratory results obtained during the design of an ultrasonic procedure that would allow dynamic characterization of local arterial situations, by applying a new technique based on continuous tracking of Power Spectral Densities (PSD) in echo-traces coming from vessel walls. Using it, a precise indication of possible geometrical deformations into those walls can be achieved under variable internal pressures with a pulsatile flow. First results show a very high potential spatial resolution (increasing with the selected overtone order). It must be noted that the applied particular autoregressive PSD technique presents an inherent good performance with noisy echo-signals, as it was reported for applications of a similar calculation algorithm to other type of spectral evaluations also into biological tissues [30–34].

In fact, one of the signal processing tools applied here is an adaptation of an algorithm included in a precise broadband ultrasonic estimation procedure intended for other previous author's applications in thermal characterization [26, 33]. Some modifications are introduced in order to be properly applied in this arterial context, for the tracking of thickness alterations in artery walls. The new technique is intended to optimize potential resolutions to detect multipulse peak frequencies in overtones received from vessels.

The proposed spectral method is novelty for this kind of vascular application. High-resolution estimators are used to detect, in the acquired complex A-scan echo-traces, possible deformations registered in the wall radial direction. A frequency domain phenomenon, “resonant” in certain way but depending on broadband pulses, often is created into many tube walls.

This is here employed for our objective because, in layers having small zones with parallel faces (e.g., in regular tube walls) appears a well-defined resonance frequency, specifically related to the distance existing between consecutive echoes coming from the wall faces, concretely, in the case of arteries, those produced by the medium-adventitia and intima-liquid interfaces.

This resonance frequency can be approximated as

(2) $$\begin{array}{c} \gamma_{1}=\frac{℧}{2*ƶ}, \end{array}$$

where *℧* is the ultrasound velocity in the artery or phantom wall and *ƶ* is the wall thickness. When this thickness augments, the said resonance frequency decreases. The variations on this value, depending on *ƶ* changes, can be described in the following way:

(3) $$\begin{array}{c} {\Delta \gamma }_{1}=-{\gamma_{o}}_{1}\left[\frac{1}{1+\left({ƶ}_{o}/\Delta ƶ\right)}\right]. \end{array}$$

In (3), *γ*_*o*__1_ is the frequency value related to an initial wall thickness, *ƶ*_*o*_, and Δ*ƶ* is the absolute deformation induced on the wall thickness.

In most of the cases, the peak of the fundamental resonance frequency (2) does not present a significant amplitude in the results of the spectral ultrasonic estimator. This is due to (often) the value of this fundamental frequency that does not fall inside the detector ultrasonic transducer band. In these cases, sufficiently high values in the harmonic order of this fundamental frequency (*γ*_1_), falling inside the nominal transducer band, must be determined. The values of all the harmonic resonant frequencies are given by

(4) $$\begin{array}{c} \gamma_{j}=j*\frac{℧}{2*ƶ}, \end{array}$$

where *j* indicates harmonic order. And, variations on *j*-harmonic value are defined as

(5) $$\begin{array}{c} {\Delta \gamma }_{j}=-{\gamma_{o}}_{j}\left[\frac{1}{1+\left({ƶ}_{o}/\Delta ƶ\right)}\right]. \end{array}$$

In (5), *γ*_*o*__*j*_ is the initial value of the *j*-harmonic related to an initial wall thickness, *ƶ*_*o*_, (i.e., the initial distance between wall interfaces).

### 2.1. Power Spectral Density Estimators

The power spectral density is defined as the discrete time Fourier transform (DTFT) of the autocorrelation sequence, *r*_ss_, of a discrete random process, *s*(*n*), that in our case represents the A-scan signal:

(6) $$\begin{array}{c} P_{\text{ss}}\left(\gamma \right)=T\overset{\infty }{\underset{m=-\infty }{\sum }}r_{\text{ss}}\left[m\right]*\exp\left(-j2\pi \gamma mT\right), \end{array}$$

where *T* is the sampling interval.

This function is a density measurement (power related to the unit of frequency) that represents the distribution of power with frequency.

In the present work, parametric PSD's estimators are used. They are characterized by their improved resolution working with short data records and their low variance. The parametric estimators are based on PSD calculation from parameters of a model rather than the autocorrelation sequence. By this way, a time-series model of the random process can be assumed; in this case, as an autoregressive (AR) process model, described by:

(7) $$\begin{array}{c} s\left[n\right]=-\overset{p}{\underset{k=1}{\sum }}a\left[k\right]s\left[n-k\right]+u\left[n\right], \end{array}$$

where *p* is known as the order of the AR model, *a*[*k*] is the *k*th model parameter, and *u*[*n*] is the “driving” sequence, supposed as white noise with zero mean and variance *ρ*_*ω*_. The PSD of the time-series model will be, then, a function of the model parameters rather than the autocorrelation sequence, defined as

(8) $$\begin{array}{c} P_{\text{AR}}\left(\gamma \right)=\frac{T\rho_{\omega }}{{\left|1+\sum_{k=1}^{p}a\left[k\right]\exp\left(-j2\pi \gamma kT\right)\right|}^{2}}. \end{array}$$

There are several methods to solve (8) for a discrete time series, *s*(*n*). In this section, two of these methods, giving high resolution (HR) in frequency domain will be briefly defined as PSD's estimators: Yule Walker and Burg methods. An extension of both will be applied in this work, to compute the PSD's (with an ultrahigh resolution) of ultrasonic waveforms containing several pulsed signals acquired from vessel walls. These calculation options are precisely chosen here because they give good results in other different applications experienced by the authors for other distinct echographic purposes developed in similar media and frequency range [35–37].

#### 2.1.1. AR Spectral Estimation by Using the Yule Walker Equations

This method is based on a relationship established between AR parameters, *a*[*k*], and the autocorrelation sequence, *r*_ss_, of the random process. This relationship can be expressed by means of AR Yule Walker equations, in matrix expression:

(9) $$\begin{array}{c} \left[\begin{array}{cccc} r_{\text{ss}}\left(0\right) & r_{\text{ss}}\left(-1\right) & \cdots & r_{\text{ss}}\left(-p\right)\\ r_{\text{ss}}\left(1\right) & r_{\text{ss}}\left(0\right) & \cdots & r_{\text{ss}}\left(-p+1\right)\\ \vdots & \vdots & \ddots & \vdots \\ r_{\text{ss}}\left(p\right) & r_{\text{ss}}\left(p-1\right) & \cdots & r_{\text{ss}}\left(0\right) \end{array}\right]\;\left[\begin{array}{c} 1\\ a{\left[1\right]}_{}\\ \vdots \\ a{\left[p\right]}_{} \end{array}\right]=-\left[\begin{array}{c} \rho_{\omega }^{2}\\ 0\\ \vdots \\ 0 \end{array}\right]. \end{array}$$

*a*[*k*] parameters can be obtained using the Levinson-Durbin algorithm [27, 28].

#### 2.1.2. AR Spectral Estimation Based on the Burg Method

Burg method assumes the approximation of a linear prediction, which supposes the minimization of the direct and inverse errors of certain linear predictors defined as

(10) $$\begin{array}{c} f_{m}\left(n\right)=s\left(n\right)-\hat{s}\left(n\right),\\ g_{m}\left(n\right)=s\left(n-m\right)-\hat{s}\left(n-m\right), \end{array}$$

where *s*(*n*) is the discrete random process, $\hat{s}(n)$ is the direct lineal prediction estimate, and *s*(*n* − *m*) is the inverse lineal prediction estimate. The minimum square error is defined as

(11) $$\begin{array}{c} \epsilon_{m}=\;\overset{N-1}{\underset{n=m}{\sum }}\left[{\left|f_{m}\left(n\right)\right|}^{2}+{\left|g_{m}\left(n\right)\right|}^{2}\right]. \end{array}$$

This error is minimized by selecting the prediction coefficients according to Levinson-Durvin Recursion [27, 28]. Once the prediction coefficients (equivalent to AR model parameters) are determined, the PSD can already be estimated.

**Figure 1:**
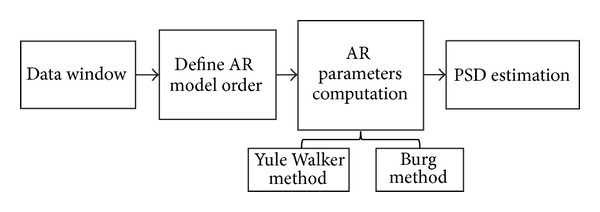
Schematic flow chart of the procedure for PSD calculation.

In Figure 1, a very schematic flow chart showing the successive phases of the spectral estimation procedure, employed here for PSD calculation, is depicted.

## 3. Materials and Methods Used for Assessing the Proposed Spectral Technique

### 3.1. Experimental Configuration for Evaluating “In Vitro” Deformations in Ducts

**Figure 2:**
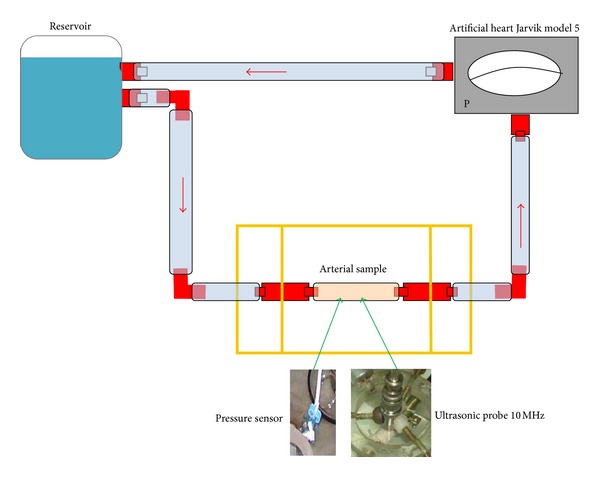
Circulating loop showing the artificial heart with a pneumatic pump, a perfusion line with the organ chamber, and a reservoir. Pressure inside the sample is obtained by using a solid state transducer. The echoes reflected from the wall faces are evaluated with a broadband ultrasonic probe (as it is explained in [24, 25]).

A circulating loop (Figure 2) was designed to make ultrasonic measurements through cross-sections of plastic tubes and arterial vessels and to take pressure signals. The circuit consists of an artificial heart coupled to a perfusion line made of polyethylene and silicon. The artificial heart (Jarvik, model 5, Kolff Medical; Salt Lake City, UT) is composed of an input valve, an output valve, and two chambers separated by a mobile diaphragm. It is driven by a pneumatic mechanical pump adapted from a mechanical respiratory device, which provides the desired heart rate, pressure values, and length of the systolic and diastolic period for each cycle.

When air is propelled, by a generator, out of the pneumatic pump, the input valve opens and the output valve closes, allowing the inflow of a fluid (saline solution) into the heart's chamber. Depending on the heart rate chosen, the corresponding time will elapse until the mechanical pump infuses air into the artificial heart. At this moment, the input valve closes and output valve opens allowing the passage of fluid into perfusion line.

The ducts samples to be tested were artery segments and tube phantoms made of latex material. Inside organ chamber a pressure sensor (Statham, with 1200 Hz in frequency response) is positioned inside the sample to measure intra-luminal pressure with 200 Hz in sampling frequency. This sensor is previously calibrated with a mercury manometer.

**Figure 3:**
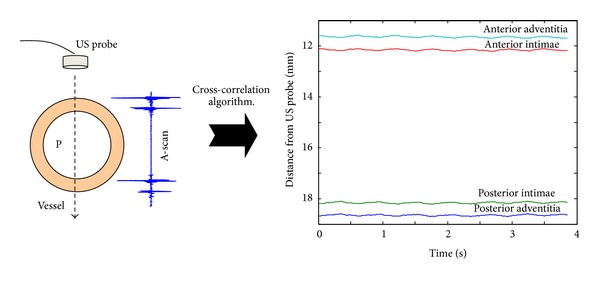
Schematic representation of the setup used for displacement measurements. Four echoes per A-scan appear, corresponding to the sample-fluid interfaces.

In order to measure the wall displacements (vertical in Figure 2), a broadband ultrasonic probe, with an useful frequency response in the range (2 to 9) MHz, is controlled in the pulse-echo mode. The experimental setup allows the positioning of the ultrasonic beam diametrically and perpendicularly to the artery wall, as it is shown in Figure 3.

Once the ultrasonic beam is set up perpendicular to the sample surface, two sets of A-scan (300 for the artery and 150 for the tube) were acquired during a number of circulating cycles at a sampling frequency of 80 MHz. In each A-scan, four echoes appear, and a first (low-resolution) simplified general display can be seen in Figure 3, where each line corresponds to each fluid-sample interface.

In this work, the following nomenclature will be used: the part of the (tube/arterial) wall which is nearer (farer) to the transducer will be called *anterior *(*posterior*) wall. For the anterior and posterior arterial walls, two echoes can be observed, one corresponds to the adventitia and the other corresponds to the intimae layer. For example, *anterior adventitia *wall will correspond to the first echo appearing in the A-scans.

Taken into account that the interest of this work is only focused on detecting local transversal changes into sections of the vessel wall and not in the general dynamic of the whole vessel, the attention will be centered exclusively in the relative displacements appearing between the two echoes belonging to each wall section. In fact, the analysis of the global dynamic and biomechanic behaviors of such an arterial segment, under the chosen experimental conditions, would be a more complex task dependent on internal and external fluids being used and also on artery clamping conditions and some spurious phenomena, which would require a more laborious previous study. In consequence, the focus of the presented ultrasonic analysis will be oriented to the inner parts of each time window associated with a specific wall section.

### 3.2. Estimation of Geometrical Deformations in Vessels Based on Cross-Correlation

Once the set of ultrasonic A-scans were acquired by using the setup of Figure 2, the internal and external vessel diameters, as a function of time throughout the acquisition interval, can be obtained as a first approach to our estimation aim of finding the relative echo positions through a time cross-correlation algorithm.

As pressure changes inside the sample, the wall position will also change. The change in the position of the wall is observed in the RF signal as a time shift (*τ*_*m*_) in the echoes produced by each wall-liquid interface. One way to estimate *τ*_*m*_ and consequently the wall-liquid interface displacement (Δ*δ*_*z*_) is to use cross-correlation. Δ*δ*_*z*_ can be estimated from *τ*_*m*_ by using the well-known pulse-echo formula:

(12) $$\begin{array}{c} {\Delta \delta }_{z}=\frac{1}{2}℧\tau_{m}, \end{array}$$

where *℧* is the ultrasound speed in the medium assumed to be 1470 m/s for the artery, 1645 m/s for the latex tube, and 1500 m/s for water.

**Figure 4:**
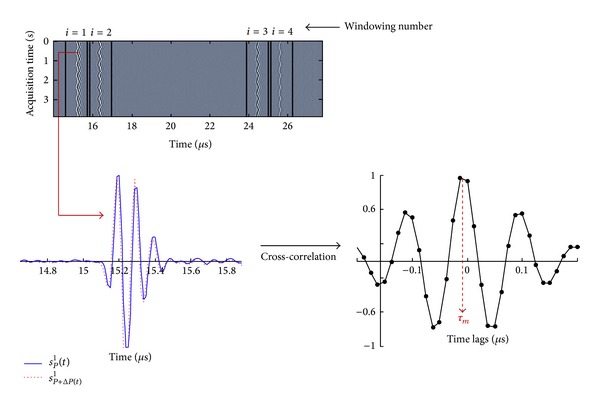
Windowing process and cross-correlation computation.

The first step to estimate *τ*_*m*_ for each wall-liquid interface consists of selecting four time windows in the RF data, each window corresponding to one echo. The center and the length of each window were chosen in a way that each echo remains within the window during the whole acquisition time. The length *T* of each window was set to 1.25 *μ*s (100 points) and 1.75 *μ*s (140 points) for the tube and the artery, respectively. As a confirmation of correct windowing, it was observed that small variations on the selections of window length and window center do not modify the final result. The whole procedure is represented in Figure 4 for the signals acquired in the tube.

Let *s*_*P*_^*i*^(*t*) be the signal corresponding to the *i*th window (*i* = 1,2, 3,4) at pressure *P*. To estimate *τ*_*m*_, the cross-correlation between *s*_*P*_^*i*^(*t*) and *s*_*P*+Δ*P*_^*i*^(*t*) is computed as follows:

(13) $$\begin{array}{c} C_{P,P+\Delta P}^{i}\left(\tau \right)=E\left(s_{P}^{i}\left(t\right)s_{P+\Delta P}^{i}\left(t+\tau \right)\right), \end{array}$$

where Δ*P* corresponds to the pressure variation between successive RF lines and *E*[] is the expectation operator. Then *τ*_*m*_ is given by the position of the maximum of *C*_*P*,*P*+Δ*P*_^*i*^(*τ*) as represented in Figure 4.

In some of the curves shown in the following sections, *τ*_*m*_ was determined more precisely, by using an added parabolic interpolation considering three points around the maximum of the correlation result. In these cases (to find thickness variations), the interpolation is performed after the estimation of the relative shifting between successive A-scans.

*Methodology Resolution and Error in the Estimation.* According to the algorithm described above, for the estimation of time shifts between successive signals, the maximum of the correlation function should be shifted by at least one sample. Consequently, the resolution of the correlation method is given by the time lag between two samples in the RF data, which in the present work is 12.5 ns (for the selected 80 MHz in the sampling frequency). Thus, based in (12), this time resolution corresponds to a 9.4 *μ*m resolution in distance. Nevertheless, if necessary, this basic resolution could be increased in some amount by the above commented parabolic interpolation, at expenses of introducing some statistical error [9, 38].

Regarding the classical delay error on time shift estimation with speckle signals, only a lower bound given by the Cramer-Rao Lower Bound (CRLB) limit can be calculated. In this work, a time shift estimation was performed using reference and delayed signals which decorrelated mainly due to a physical process (i.e., change of pressure inside the sample), which is just the effect to be measured, but both signals could also be slightly decorrelated due to the presence of jitter. Jitter occurs when noise and finite window lengths cause a slight displacement of the true peak in the cross-correlation function. When reference and delayed signals are relatively similar, the standard deviation of jitter errors for any unbiased estimator can be predicted using the CRLB. According to Walker and Trahey (1995) and Carter (1987), the standard deviation of the jitter for any unbiased delay estimator [39, 40] is given by

(14) σ(τm−τ^m) ≥1T∫−∞∞(2πf)2(χP,P+ΔPi(f)/(1−χP,P+ΔPi(f)))df,

where *τ*_*m*_ is the true time shift, ${\hat{\tau }}_{m}$ is the estimated time shift, *T* is the window length, and *χ*_*P*,*P*+Δ*P*_^*i*^(*f*) is the magnitude-squared coherence (MSC) defined as

(15) $$\begin{array}{c} \chi_{P,P+\Delta P}^{i}\left(f\right)=\left|\frac{{\tilde{C}}_{P,P+\Delta P}^{i}\left(f\right)}{{\tilde{C}}_{P,P}^{i}\left(f\right)\cdot {\tilde{C}}_{P+\Delta P,P+\Delta P}^{i}\left(f\right)}\right|, \end{array}$$

where ${\tilde{C}}_{P,P+\Delta P}^{i}\left(f\right)$ is the cross-power spectrum of the cross-correlation function defined in (13).

To estimate *χ*_*P*,*P*+Δ*P*_^*i*^(*f*), the Welch method with a window length of 0.5 *μ*s (40 points) and a 50% of window overlapping was used. For the tube and the artery cases, both parameters were kept the same. There are as many magnitude square coherences as cross-correlations functions. In this work, the magnitude square coherence used in (14) to estimate CRLB was the average of all magnitude square coherences computed from each cross-correlation function.

A CRLB estimation of 0.33 ns and 0.47 ns were obtained for the tube and artery cases, respectively, which corresponds to errors on wall displacement estimation of 0.25 *μ*m and 0.36 *μ*m, respectively, which can be neglected in comparison to resolution limits.

### 3.3. Applying Our AR Spectral Technique to HR Estimation of Wall Deformations

A set of 300 multipulse ultrasonic signals were acquired from both cases: a healthy portion in a carotid artery and latex tube was segmented in order to extract two data windows in each one, to perform the spectral analysis related to each wall, the anterior and the posterior. The procedure for ultrasonic signals acquired from artery is detailed in this section, but it was the same for the latex tube. Each window contains two echoes produced by the interfaces between (a) external liquid and arterial wall and (b) arterial wall and inner liquid, so that the times-of-flight between echoes can be related to the walls thicknesses.

**Figure 5:**
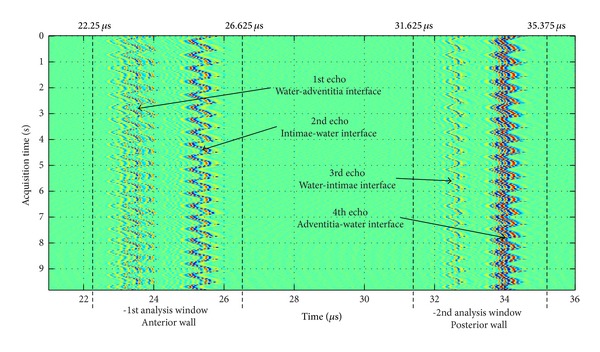
Echoes from a healthy arterial segment acquired during pump-simulated heart beats (with an acquisition interval of 9.8 s), from anterior and posterior walls.

An example of the windowing procedure, in Figure 5 is shown: a first data-window (anterior wall) established from 22.250 to 26.625 *μ*s and a second one (posterior wall) from 31.325 to 35.375 *μ*s. The periodic time displacement (due to the circulatory mechanical movement in artery) on interface echoes can be clearly appreciated, and the echoes time behavior, at the four interfaces, is illustrated during an acquisition interval of 9.8 s (signals were acquired in steps of *≈*0.030 s).

Once, data windows were detached from original signals, each signal segment was processed, to obtain its PSD by means of the two implemented parametric procedures (Yule Walker and Burg) described in Section 2.

The PSD's were estimated and analyzed to obtain, from each one, those peak values related to the harmonics of the resonance fundamental frequencies, in the vessel walls that fall inside the transducer band used for ultrasonic detection. These resonances are related to the distinct dynamically variable wall thicknesses (just, a half of the distance between the two successive echoes reflected from the same wall). The results to be obtained, for each window, are the frequency shifts on the resonant harmonics (located into the transducer band) related to the changing behavior in the separation between wall echoes for the specified acquisition time interval.

**Figure 6:**
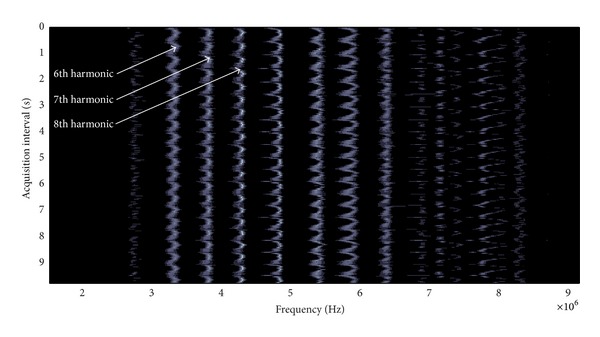
PSD's estimated using the Burg option for the first analysis window, in the signals acquired in a healthy artery wall.

As calculation example, Figure 6 shows a global display of PSD's values for the signal segments corresponding to the first analysis window obtained in this case with the version of our spectral procedure based on Burg method. Several resonant harmonic peaks, into the transducer band, can be appreciated, as well as their periodic displacements (shifts) along the whole acquisition time interval (0–9.8 s). Taking as initial wall thickness (1.32 mm), the value calculated from the first multipulse ultrasonic signal of the acquisition interval and supposing an ultrasound velocity on artery walls *℧* = 1470 m/s, a theoretical fundamental frequency initial value *γ* equal to 556.82 KHz is estimatedby using (2). Specifically, harmonics (overtones) 6th, 7th, and 8th were marked in this figure. The displacement of these harmonics is clearly of periodical type, and it is tuned with the changes in echoes separation. This behavior will be analyzed hereinafter, within the presented results.

A specific harmonic, located into the used operative transducer band, was selected in order to process the deviations in its peak value and, in this way, estimate the corresponding thickness variations of the artery wall by means of (5).

*Statistical Properties for Peak Frequency Estimation of AR Spectral Analysis.* There are several works [41–44] where the statistical fluctuations of a peak frequency estimated by means of an AR spectral estimator have been investigated. On [41], it is inferred numerically that the variance of frequency estimation is proportional to *N* ^−1^ · *β* ^−2^, where *N* is the data length and *β* is the signal to noise ratio. A simplified expression for the variance produced by these fluctuations, under the assumption that the time series *s*(*n*) consists of the sum of *p* sinusoids and zero mean stationary Gaussian noise was obtained as

(16) $$\begin{array}{c} E\left[\left({\Delta w}^{2}\right)\right]\approx \alpha \frac{\beta^{-2}}{N}, \end{array}$$

where *w* is the angular frequency and *α* is a constant which depends on *w* and the model order *p*. Also, in this work, experimental graphs show that if *βp* ≫ 1, the estimator is unbiased.

Based on this formulation, it is possible to assume that the AR spectral estimators used to process signals in this work (considering *N* = 200, *p* = 90, and *β* > 20 dB) are unbiased estimators with a small variance; however, as no analytical justification is established in the literature, a specific experimental analysis focused to variance estimation has to be carried out in the future.

## 4. Results Evaluation for PSD Estimation of Deformations on Wall Thickness

In order to show the good performance of the proposed PSD estimation technique the following is required.

- First, comparative analyses are made between results calculated for the geometrical deformations registered in the phantom (latex tube) and carotid artery segment, which were obtained from experimental echo-traces and alternatively using (i) Classical Time-Cross-Correlation (TCC) and (ii) Proposed AR Parametric Spectral Estimation.
- Finally, more extended results of the new PSD technique, proposed here for ultra-high-resolution spectral estimation are shown, when it was applied in both ducts (of latex and arterial tissue). Spatial (thicknesses and deformations) and frequency curves are included.

### 4.1. Comparisons in Wall Geometrical Deformations Obtained with Classical TCC and the Proposed PSD Methods

In order to make a comparison in rather repetitive laboratory conditions, a phantom constructed with a tube made of latex material was taken as a duct of reference.

The two techniques, one based on the new spectral estimation here proposed and other based on a typical cross-correlation operator were applied to estimate deformations created in the walls of ducts, under changing pressures in internal flowing liquid.

**Figure 7:**
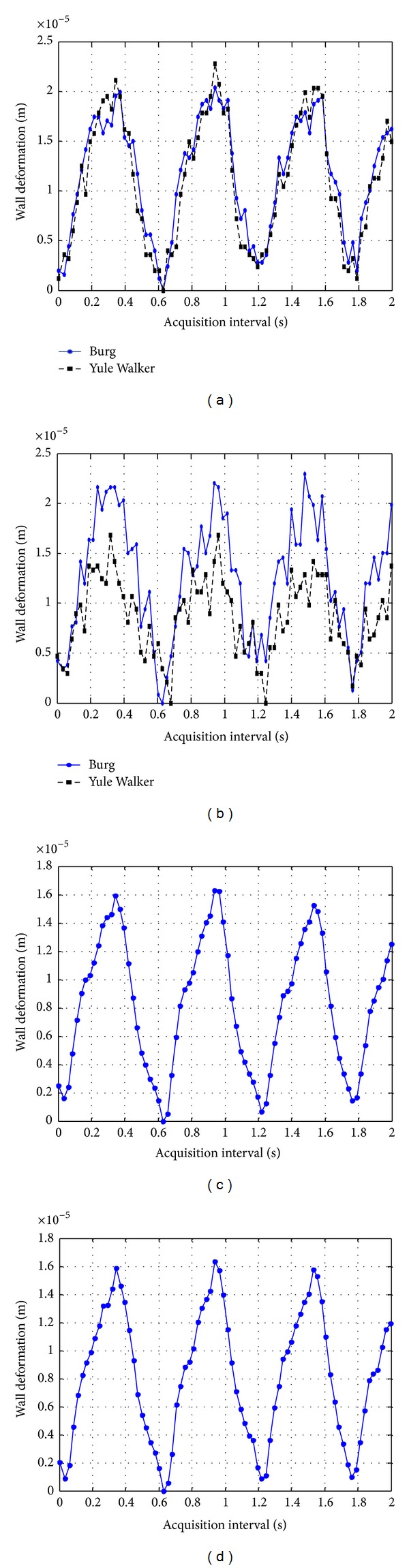
Results of dynamic thickness deformation in the two walls of a latex tube obtained with two PSD methods for (a) anterior and (b) posterior face; and with TCC processing for (c) anterior and (d) posterior face.

*Comparison Results in a Latex Tube.* Figure 7 shows anterior and posterior wall deformation estimates obtained with both techniques in a 2-second time window. In the case of spectral (PSD) technique, results obtained with Burg and Yule walker methods are graphed.

The maximum wall deformations for the latex tube are listed on Table 1. Deformation estimates with both techniques, TCC and PSD, have similar values. The minimum difference between results was obtained between estimations from TCC and Burg methods for the anterior wall, which is just of 3 *μ*m (approximately 30% of TCC resolution value of 9,4 *μ*m) and for the posterior wall, the minimum difference was obtained between TCC and Yule Walker methods; it was 0.5 *μ*m (aprox. 5% of TCC resolution value).

On the other hand, it is important to mention that in order to reach a convenient resolution for the TCC method (with an initial resolution of 9.4 *μ*m) to be compared with spectral methods (with a resolution of 399 nm considering a frequency resolution of 2.44 kHz on the seventh harmonic), a parabolic interpolation for TCC was applied, though this interpolation could increase the method bias [9].

**Figure 8:**
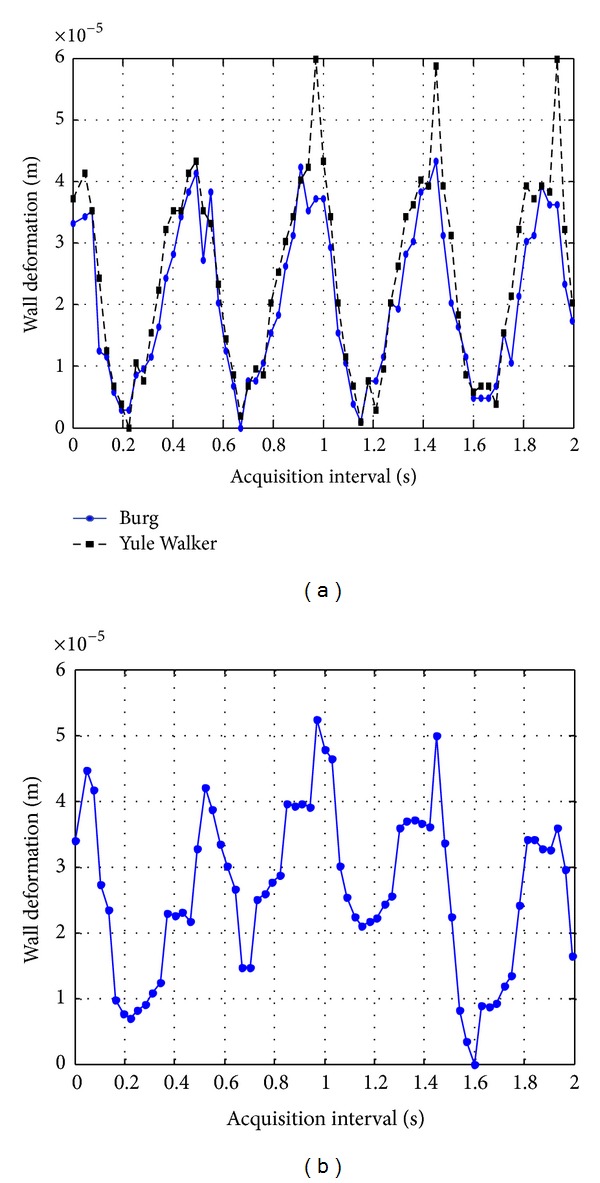
Results of dynamic thickness deformation in anterior wall face of a carotid segment with two PSD processing (a) and with TCC processing (b).

*Comparison Results “In Vitro” on a Carotid Artery.* In Figure 8, anterior wall deformation estimates obtained for the carotid artery by means of two methods are depicted. In this illustration a time window of 2 seconds was used too.

The maximum wall deformations for the carotid artery are listed in Table 2. In this case a minimum difference of 9 *μ*m was encountered between TCC and spectral methods.

As in the previous case, resolution of TCC method was improved by means of parabolic interpolation to be compared with the resolution of spectral methods reached by the artery case (963 nm considering a 2.44 kHz of frequency resolution on the sixth harmonic).

**Figure 9:**
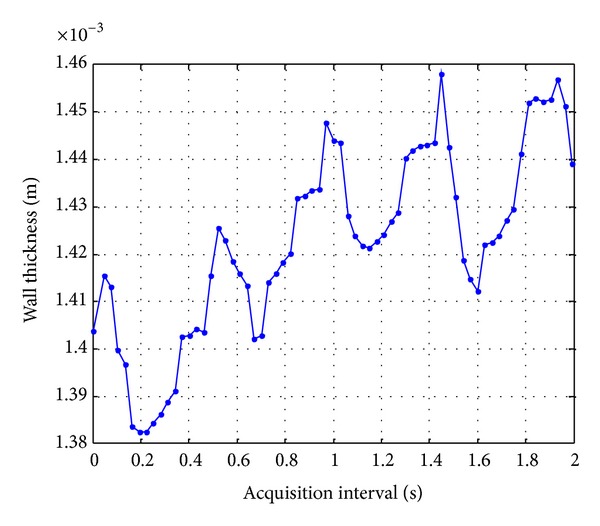
Wall deformation estimates obtained by means of TCC method before removing the linear trend.

Also, in this case of the carotid artery, wall deformation values estimated with TCC method appear to be superposed over a positive slope baseline, as can be observed in Figure 9. Data has to be processed, in order to remove these trend and obtain the wall deformation estimates shown in Figure 8(b) to be compared with estimates produced with spectral methods.

The obtained results in this analysis shows that the proposed spectral techniques estimate wall deformation values with high accuracy (resolution in the micrometric order), and avoiding some current problems presents in the TCC method, as the trend (baseline) presented in Figure 9.

### 4.2. Spatial and Frequency Results of the Ultra-High-Resolution Spectral Estimation in Phantom and Carotid Segment

**Figure 10:**
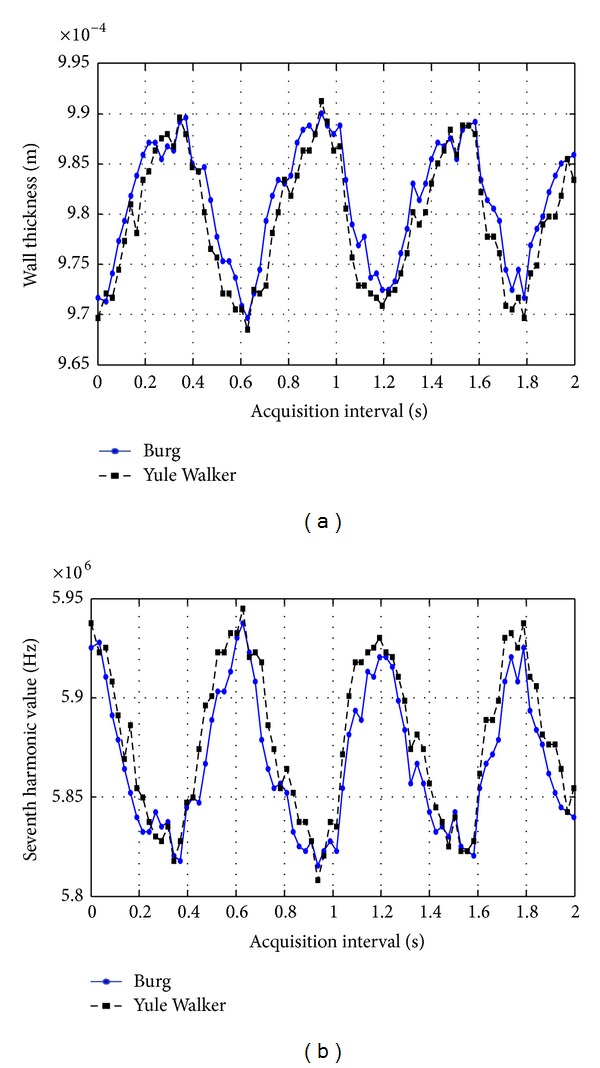
Behaviour of wall thickness (a) and 7th overtone frequency (b) for the latex tube.

*Results in the Latex Tube.* In Figure 10, specific results obtained by means of spectral methods, Burg and Yule Walker applied to signals acquired from the latex tube are graphed. The anterior wall thickness behaviour is shown, which is varying between 0.97 and 0.99 mm. Also, the corresponding variations (between 5.81 and 5.95 MHz) on the seventh harmonic value, which were used to estimate wall thickness values by means of (5), are graphed. It is illustrated that a decrease of the selected harmonic represents an increase of the wall thickness, as described in Section 2. The resolution defined to compute PSD's was 2.44 kHz, which represents, for the seventh harmonic and considering a velocity of 1645 m/s for the latex tube wall, a resolution of 399 nm.

*Results in the Carotid Artery.* The sixth harmonic of the PSD's, estimated from the first data window (anterior wall) of the sequence of multipulse signals acquired in a healthy artery, was selected to analyze its behavior and related it to the measured wall thickness variations. The expected peak frequency value was calculated on 3.34 MHz for this harmonic, where PSD's were estimated with a resolution of 2.44 kHz, which represents (in the spatial domain) a still excellent potential thickness resolution of around 963 nanometers, for the considered 6th overtone of the wall internal resonance here.

**Figure 11:**
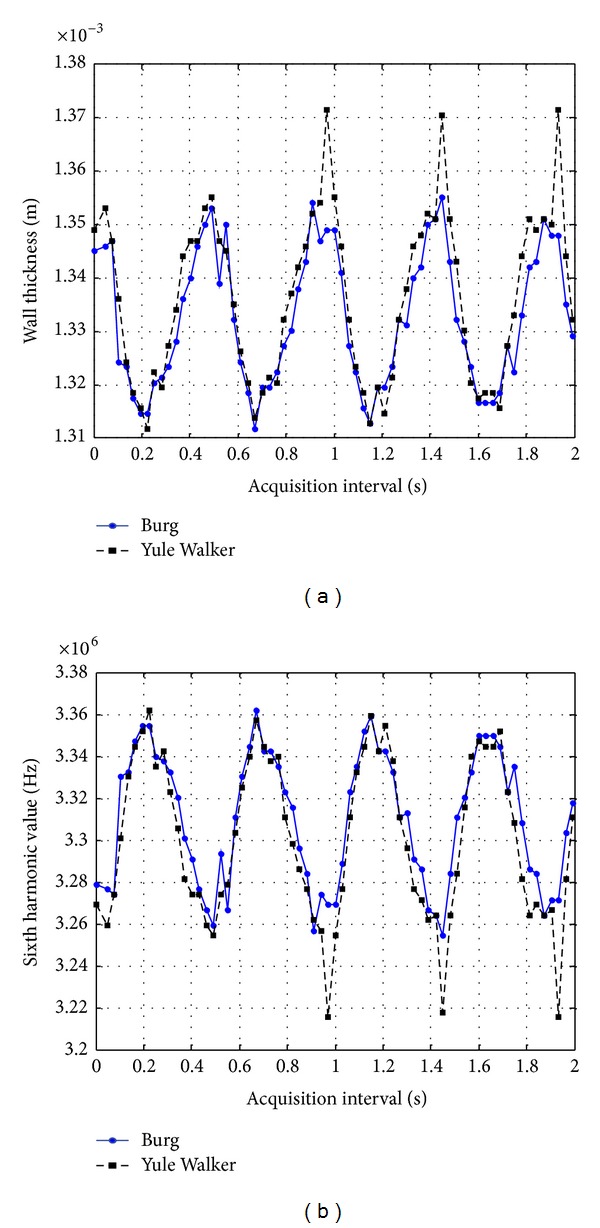
Resulting wall thickness values of the anterior wall of carotid artery (a) calculated from 6th harmonic values (b).

Figure 11 shows the periodical variation of the peak frequency in the 6th harmonic obtained from the PSD's calculated for the first 2 seconds of the acquisition interval. The peak value for the 6th harmonic equal to 3.34 MHz, corresponds to a reference wall thickness of 1.32 mm. Two graphics are displayed, the blue one corresponds to values obtained with Burg algorithm and the black curve corresponds to the values obtained with the Yule Walker option.

Also in this figure, the corresponding wall thickness variations are shown for this case calculated from (5) and based on the sixth harmonic values presented in the right side. From an reference value for wall thickness of 1.32 mm, a maximum thickness variation of approximately 35 *μ*m is detected with Burg and Yule Walker algorithms. On the other hand, minimum thickness variations smaller than 10 *μ*m were estimated also with both options.

Analyzing the behavior of these graphics, the relation between harmonic peak values and thickness variation can be clearly observed too for the case of the artery: a harmonic value increment corresponds to a reduction in wall thickness. Quite the opposite, when harmonic value diminishes, the wall thickness increases.

**Figure 12:**
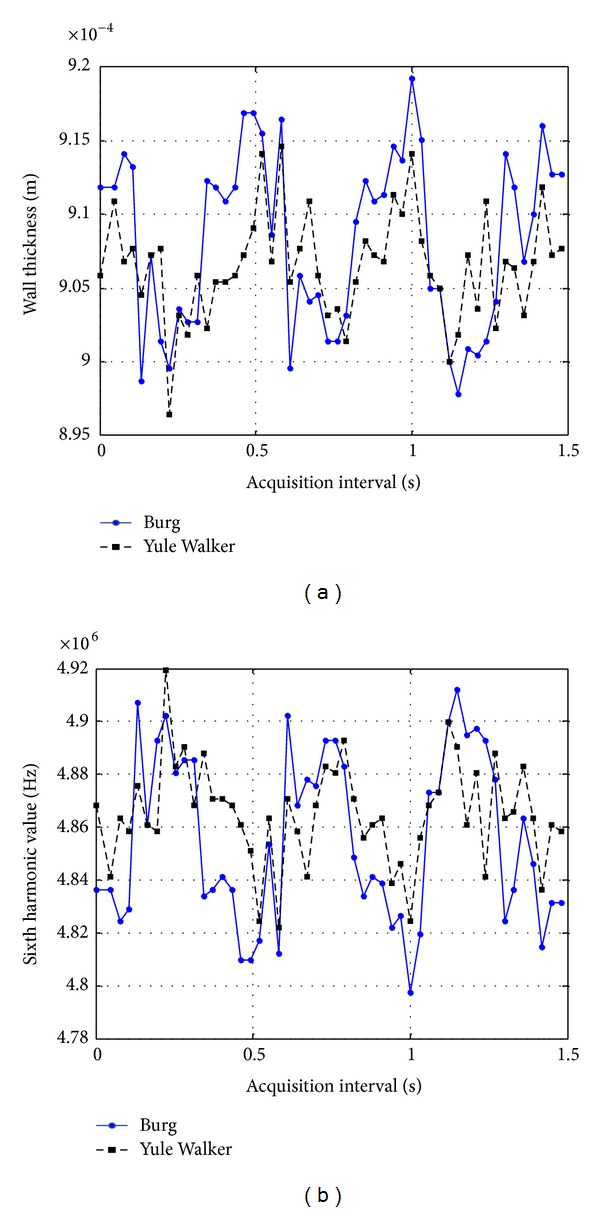
Resulting wall thickness values of the posterior wall of carotid artery (a) calculated from 6th harmonic values (b).

In Figure 12, the posterior wall thickness variations are presented and the related sixth harmonic values are also graphed. In this wall, thickness varies between a minimum value of 0.897 mm and a maximum value of 0.919 mm, and the corresponding 6th harmonic values varies between a maximum value of 4.92 MHz and a minimum value of 4.79 MHz. The difference between the minimum and maximum thickness value is 22 *μ*m, as reported in Table 2 for the posterior wall.

**Figure 13:**
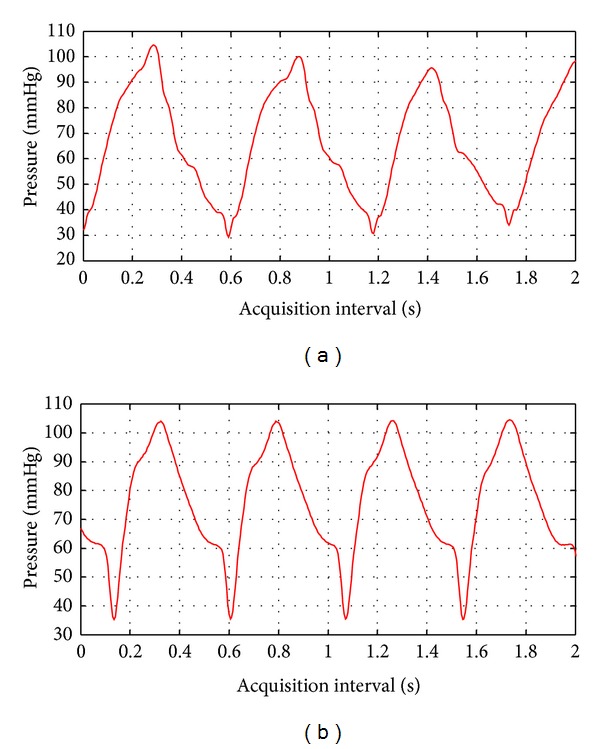
Pressure variations during an acquisition interval of 2 seconds, for latex tube (a) and carotid artery (b).

As mentioned before, the experimental setup was driven by a mechanical respiratory device, which provides the desired heart rates and the pressure values for each case, latex tube phantom and carotid artery. In Figure 13, the pressure variations applied in both cases are shown for an acquisition interval of 0 to 2 s.

## 5. Discussion of Results

Wall thickness variations for carotid artery segments were estimated in previous section by using two options: a cross-correlation based method TCC and a new improved spectral procedure PSD. It can be observed that the thickness and deformation values of the walls, illustrated on previous figures (obtained with correlation and spectral options for the case of a healthy anterior wall), are varying coherently with the pressure behavior in Figure 13. When the pressure value augments, the wall thickness diminishes and conversely as expected. The posterior wall thickness of this healthy artery varies in a similar manner and with the same cadence, as shown in Figure 12 for our spectral procedure.

Due to mechanical properties of major clinical interest are based mainly on the thickness variation value (relative to a reference threshold), and not on the estimation of an absolute wall thickness value; it must be considered that an efficient estimator has to provide the most precise results for the thickness variations, because it gives direct information about the deformations suffered by the wall artery under variable internal pressures. Additionally, during elasticity calculation processes, it is the required information. In this regard, our spectral procedure shows a good performance about the thickness variations measurements and their uniformity, accordingly to pressure changes; this means that all the maximum and minimum thickness values are located around a mean fixed value, while the results obtained by means of correlation, are not varying in this convenient way.

As other index of accuracy in this type of evaluation techniques, it must be noted that the wall thickness resolution obtained by means of the spectral procedures proposed here could arrive to 963 nm (as mentioned above, by considering a spectral resolution of 2.44 kHz) when the 6th harmonic was analyzed. Besides, it must be taken into account that this already high resolution may be still grown, potentially, by applying two optional paths: (a) increasing the frequency step chosen, but this has a limit in the practice, and a specific analysis would have to be made to establish an accepted threshold in the said step for this kind of estimations and (b) analyzing a larger value in the order of the echoes harmonic being analyzed, because the variation produced in the harmonic peak frequency by a specific thickness change is directly proportional to the harmonic order, *j*, as can be seen in (4) and (5); that is, the variation in the *j*th harmonic will be larger than in the *j*th−1 case. And for this reason, the same absolute value in spectral resolution will represent a higher spatial resolution for the wall thickness measure, when the *j*th harmonic instead of the *j*th−1 is used.

Nevertheless, there is a clear limit in attempting any possible ulterior improvement in resolution, which is always related to the signal-to-noise ratio (SNR) encountered in real medical applications. In this sense, the paper authors already have analyzed, previously [30, 32–34], the influence of possible poor SNR levels in ultrasonic echoes on the induction of errors in results of our spectral estimation technique (AR-PSD). Simulation results during these computational analyses indicate that the AR-PSD processing option presents an inherent good performance with noisy echo-signals, as it was proved in the practice by applications of our calculation algorithms to other spectral evaluations also into tissues. In fact, SNRs very low (up to only 1 dB) were studied, and as a general result, if the echo-signals have a SNR better than 3 dB (which is easily attained in the usual bio-ultrasonic measurements), the error induced on our spectral PSD estimation uses a range around a value of 0,1%, which represents frequency steps similar to those used in the calculation presented in this paper (2,44 kHz).

## 6. Conclusions

An ultra-high-resolution spectral technique working in echo overtones range, improved to assure accurate measurements of arterial wall deformations, was proposed. It can be used to provide critical data for general wall evaluation and for elastographic purposes. Burg and Yule Walker algorithms were alternatively implemented as two interesting options in a calculation step integrated in the proposed technique.

In order to calibrate the potential performance of this technique, two processing options were applied to evaluate wall thickness and its deformation during a circulatory cycle: a typical time cross-correlation operator (TCC) and the proposed procedure (PSD). An experimental setup, simulating heart systolic/diastolic periods, was used to inspect a latex phantom and samples of a carotid artery. As example, the results in the phantom, TCC-based method shows a mean deformation value for the wall thickness of 16,5 microns in anterior wall, whereas the proposed spectral technique gives a value of 19,5 microns. This little difference is quite smaller than the basic resolution in the TCC technique (9,4 microns). And in the arterial case, the deformation values for anterior wall were 36 and 41 microns, also with a difference smaller than the TCC resolution.

In general, both processing options estimate wall thickness variations in accordance with the periodic changes in internal pressure; nevertheless, the results with the basic cross-correlation operator are not centered around a stable baseline and it would be needed to make some type of interpolation or detrend. This effect could be produced by secondary complex movements of the whole artery, additional to the radial displacements of interest. Spectral option results suggest that this technique has a special robustness against this type of perturbations by artery movements.

Regarding the resolution capability of the new estimation technique, submicrometric values were achieved in the first experiments, with frequency steps of 2,44 kHz and harmonic orders of 6 and 7, but further computational analyses must be made in order to investigate resolution limits in function of both parameters and under distinct levels of noise induced in the echoes. In addition, the effects of these thresholds must be confirmed in the practice, into tissues and under more realistic working conditions.

And, as other future research works, the influence of processing aspects and conditions, like interpolation tools, type of windowing, and the overtone order being considered, could be analyzed. The final aim of the work would be to confirm the achieving of ultra-high spatial resolutions, penetrating into the nanometric order.

## Figures and Tables

**Table 1:** Maximum wall deformations estimated for latex tube.

Anterior wall	
TCC	16,5 µm
Spectral estimation Burg	19,5 µm
Spectral estimation Yule-Walker	21,3 µm

Posterior wall	

TCC	16,5 µm
Spectral estimation Burg	22,0 µm
Spectral estimation Yule-Walker	17,0 µm

**Table 2:** Maximum wall deformations estimated for carotid artery.

Anterior wall	
TCC	51 µm
Spectral estimation Burg	42 µm
Spectral estimation Yule-Walker	60 µm

Posterior wall	

TCC	— µm
Spectral estimation Burg	22,0 µm
Spectral estimation Yule-Walker	18,0 µm

## References

[1] Anderson KM, Odell PM, Wilson PWF, Kannel WB (1991). Cardiovascular disease risk profiles. *American Heart Journal*.

[2] De Backer G, Ambrosioni E, Borch-Johnsen K (2003). European guidelines on cardiovascular disease prevention in clinical practice: third Joint Task Force of European and other Societies on Cardiovascular Disease Prevention in clinical practice. *European Heart Journal*.

[3] Simon A, Levenson J (2005). May subclinical arterial disease help to better detect and treat high-risk asymptomatic individuals?. *Journal of Hypertension*.

[4] Bonnefous O, Pesqué P (1986). Time domain formulation of pulse-Doppler ultrasound and blood velocity estimation by cross correlation. *Ultrasonic Imaging*.

[5] Hoeks APG, Arts TGJ, Brands PJ, Reneman RS (1993). Comparison of the performance of the RF cross correlation and Doppler autocorrelation technique to estimate the mean velocity of simulated ultrasound signals. *Ultrasound in Medicine and Biology*.

[6] Hansen F, Mangell P, Sonesson B, Länne T (1995). Diameter and compliance in the human common carotid artery—variations with age and sex. *Ultrasound in Medicine and Biology*.

[7] Brands PJ, Willigers JM, Ledoux LAF, Reneman RS, Hoeks APG (1998). A noninvasive method to estimate pulse wave velocity in arteries locally by means of ultrasound. *Ultrasound in Medicine and Biology*.

[8] Hasegawa H, Kanai H, Hoshimiya N, Chubachi N, Koiwa Y (1998). Accuracy evaluation in the measurement of a small change in the thickness of arterial walls and the measurement of elasticity of the human carotid artery. *Japanese Journal of Applied Physics 1*.

[9] Rabben SI, Bjærum S, Sørhus V, Torp H (2002). Ultrasound-based vessel wall tracking: an auto-correlation technique with RF center frequency estimation. *Ultrasound in Medicine and Biology*.

[10] Kanai H, Hasegawa H, Ichiki M, Tezuka F, Koiwa Y (2003). Elasticity imaging of atheroma with transcutaneous ultrasound: preliminary study. *Circulation*.

[11] Hasegawa H, Kanai H, Koiwa Y (2002). Modified phased tracking method for measurement of change in thickness of arterial wall. *Japanese Journal of Applied Physics 1*.

[12] Catheline S, Wu F, Fink M (1999). A solution to diffraction biases in sonoelasticity: the acoustic impulse technique. *Journal of the Acoustical Society of America*.

[13] Bazán I (2009). *Evaluation and improvement of ultrasonic techniques to non-invasive estimation of internal thermal distributions in biological phantoms with scatterers [Doctorate thesis]*.

[14] Armentano R, Megnien JL, Simon A, Bellenfant F, Barra J, Levenson J (1995). Effects of hypertension on viscoelasticity of carotid and femoral arteries in humans. *Hypertension*.

[15] O’Donnell M, Skovoroda AR, Shapo BM, Emelianov SY (1994). Internal displacement and strain imaging using ultrasonic speckle tracking. *IEEE Transactions on Ultrasonics, Ferroelectrics, and Frequency Control*.

[16] Kanai H, Hasegawa H, Chubachi N, Koiwa Y, Tanaka M (1997). Noninvasive evaluation of local myocardial thickening and its color-coded imaging. *IEEE Transactions on Ultrasonics, Ferroelectrics, and Frequency Control*.

[17] Kanai H, Koiwa Y, Zhang J (1999). Real-time measurements of local myocardium motion and arterial wall thickening. *IEEE Transactions on Ultrasonics, Ferroelectrics, and Frequency Control*.

[18] Hasegawa H, Kanai H, Hoshimiya N, Koiwa Y (2004). Evaluating the regional elastic modulus of a cylindrical shell with nonuniform wall thickness. *Journal of Medical Ultrasonics*.

[19] Inagaki J, Hasegawa H, Kanai H (2005). Construction of reference data for tissue characterization of arterial wall based on elasticity images. *Japanese Journal of Applied Physics*.

[20] Hasegawa H, Kanai H (2009). Strain imaging of arterial wall with reduction of effects of variation in center frequency of ultrasonic RF echo. *FMBE Proceedings*.

[21] Hasegawa H, Kanai H (2006). Improving accuracy in estimation of artery-wall displacement by referring to center frequency of RF echo. *IEEE Transactions on Ultrasonics, Ferroelectrics, and Frequency Control*.

[22] Hasegawa H, Kanai H (2006). Modification of the phased-tracking method for reduction of artifacts in estimated artery wall deformation. *IEEE Transactions on Ultrasonics, Ferroelectrics, and Frequency Control*.

[23] Brum J, Bala G, Bia D, Armentano R, Negreira C Accuracy measurement of the arterial wall elasticity using an ultrasonic speckle correlation technique.

[24] Brum J, Balay G, Bia D (2010). Improvement of Young modulus estimation by ultrasound using static pressure steps. *Physics Procedia*.

[25] Brum J, Bia D, Benech N (2010). Set up of a cardiovascular simulator: application to the evaluation of the dynamical behavior of atheroma plaques in human arteries. *Physics Procedia*.

[26] Bazán I, Vazquez M, Ramos A, Vera A, Leija L (2009). A performance analysis of echographic ultrasonic techniques for non-invasive temperature estimation in hyperthermia range using phantoms with scatterers. *Ultrasonics*.

[27] Proakis JG, Manolakis DG (1998). *Digital Signal Processing*.

[28] Marple SL (1987). *Digital Spectral Analysis with Applications*.

[29] De Jong PGM, Arts T, Hoeks APG, Reneman RS (1990). Determination of tissue motion velocity by correlation interpolation of pulsed ultrasonic echo signals. *Ultrasonic Imaging*.

[30] Ramos A, Bazán I, Negreira C (2012). Estimation of PSD shifts for high-resolution metrology of thickness micro-changes with possible applications in vessel walls and biological membrane characterization. *Sensors*.

[31] Bazán I, Ramos A, Ramirez A, Castellanos L, Posadas R Valuation of responses in spectral techniques for thermal estimation into biological media, from ultrasonic echo-signals contaminated with increasing noise levels.

[32] Ramos A, Negreira C, Bazan I, Brum J Detecting microchanges in thin thickness of latex duct walls with 10 MHz ultrasound pulses.

[33] Bazan I, Ramos A, Calas H (2012). Possible patient early diagnosis by ultrasonic noninvasive
estimation of thermal gradients into tissues based on spectral
changes modeling. *Computational and Mathematical Methods in Medicine*.

[34] Hipólito V, Luna S, Bazán I Multi-echo signals simulation based on a mathematical model adjusted to hepatic tissue echographic behavior.

[35] Bazán I, Ramos A, Ramirez A, Castellanos L Computational evaluation of the thermal resolution on ultrasonic thermometry using an improved spectral analysis of echo-signal patterns modeled ad-hoc.

[36] Bazan I, Ramos A, Trujillo L Quantitative predictive analysis of responses obtained from a strictly noninvasive procedure based on parametric PSD estimation for measurements of thermal properties inside biological tissues.

[37] Ramos A, Bazan I, Negreira C, Brum J, Rosales A, Gallegos F Analyzing wall thickness of artery phantoms in a noninvasive way.

[38] Moddemeijer R (1991). On the determination of the position of extrema of sampled correlators. *IEEE Transactions on Signal Processing*.

[39] Walker WF, Trahey GE (1995). Fundamental limit on delay estimation using partially correlated speckle signals. *IEEE Transactions on Ultrasonics, Ferroelectrics, and Frequency Control*.

[40] Carter GC (1987). Coherence and time delay estimation. *Proceedings of the IEEE*.

[41] Sakai H (1979). Statistical properties of AR spectral analysis. *IEEE Transactions on Acoustics, Speech, and Signal Processing*.

[42] Keeler RJ (1978). Uncertainties in adaptive maximum entropy frequency estimators. *IEEE Transactions on Acoustics, Speech, and Signal Processing*.

[43] Swingler DN (1980). Frequency errors in MEM processing. *IEEE Transactions on Acoustics, Speech, and Signal Processing*.

[44] Thorvaldsen T (1981). A comparison of the least squares method and the burg method for autoregressive spectral analysis. *IEEE Transactions on Antennas and Propagation*.

